# The Importance of Root Interactions in Field Bean/Triticale Intercrops

**DOI:** 10.3390/plants9111474

**Published:** 2020-11-02

**Authors:** Dayana N. Esnarriaga, Marco Mariotti, Roberto Cardelli, Iduna Arduini

**Affiliations:** 1 Department of Agricultural, Food and Environmental Sciences, University of Pisa, Via del Borghetto 80, 56124 Pisa, Italy; d.esnarriaga@studenti.unipi.it (D.N.E.); roberto.cardelli@unipi.it (R.C.); 2 Department of Veterinary Sciences, University of Pisa, Viale delle Piagge 1, 56124 Pisa, Italy; marco.mariotti@unipi.it

**Keywords:** field bean, intercrops, legume/cereal, N level, nodule, P facilitation, root interactions, root morphometry, soil enzyme activity, triticale

## Abstract

To highlight the contribution of belowground interactions to biomass and N and P yields, field bean and triticale were grown in a P-poor soil as sole crops and as replacement intercrops at two N levels. The shoots were always in contact, while the roots of adjacent rows were free to interact or were completely separated. This allowed simultaneous testing the intraspecific and interspecific competition between rows, which to our knowledge has not been studied before. Root biomass, distribution in soil, morphometry, and functional traits were determined, together with the nodule number and biomass. The Land Equivalent Ratio for shoot biomass and N and P yield were higher than 1 when roots were in contact, and markedly lower when they were separated. This demonstrates the positive contribution of root interactions, which in field bean, consisted of increased root elongation without changes in biomass and nutrient status; in triticale, of increased N and P uptake efficiency and reduced biomass partitioning to roots. The soil-plant processes underlying intercrop advantage led to complementarity in N sources with low N inputs and facilitated N and P uptake with high N inputs, which demonstrates that intercropping could be profitable in both low and high input agriculture.

## 1. Introduction

The ecological intensification of crop production improves the environmental and economic sustainability of agricultural systems by better exploiting the ecological processes occurring in plant-soil systems, thus obtaining comparable yields to industrial agriculture but with lower external inputs [1,2]. Intercropping is the coexistence of two or more crops in the same field at the same time. It is a key strategy in ecological intensification, as it is hypothesized that the complementarity in resource acquisition of the mixed species better exploits the environmental resources [3]. Interspecific interactions have also been found to increase the mobilization and phytoavailability of limiting soil nutrients, such as phosphorus, iron, and zinc, through the exudation of organic compounds and changes in the pH of the rhizosphere [4,5]. Intercrops have also been found to positively influence soil biota by reducing the development of specialized pathogens, which are detrimental especially to graminoid monocrops [6,7]. Intercropping systems therefore would seem to promote productivity, stability of outputs, and resilience to disturbance [3].

*Poaceae* (grasses) and *Fabaceae* (legumes) are frequently intercropped because they are complementary components in livestock diets and also because of their well-known complementarity in the use of nitrogen sources, which relies on the ability of legumes to establish a symbiosis with atmospheric-N fixing bacteria [8,9]. Besides that, grass and legume crops differ in terms of the morphology and distribution of roots and promote different soil microorganisms [10,11]. This could reduce the competition for water and mineral acquisition in intercrops compared to sole crops, increase the diversity of soil biota, and promote facilitation processes, leading to a better exploitation of soil resources [2,12]. Despite the basic structural differences between the roots of grasses and legumes, intercropping has been found to change the overall root distribution and architecture by favoring the development of different types of roots, and also affects exudation processes in the rhizosphere [1,13]. In cowpea/maize intercrops, Latati et al. [14] found a higher concentration of available P in the rhizosphere of both species, which was associated with the acidification of the rhizosphere around legume roots. Besides direct facilitation, which is mediated by the acidification caused by biological N_2_-fixation, indirect microbe-mediated facilitation and niche complementarity also appear to enhance the acquisition of P, Fe, and Zn in cereal/legume intercrops [15]. Liu et al. [16] also reported that the exudation of flavonoids by wheat roots promotes nodulation in faba bean, thus supporting complementarity in the use of N sources.

The biomass yield of intercrops compared to sole crops, together with their response to N levels, are priority goals in intercropping research [17,18,19,20]. However, to ensure the higher yields of intercrops, crop density is often set higher than in sole crops, which increases the competition for light of aerial plant parts, favoring or limiting one of the partner species, especially when they differ in size and growth patterns [14,21,22,23]. Competition always has an energy cost for plants, which reduces the performance of at least one partner crop and, generally, makes them more prone to diseases [2,18]. Interspecific interactions in intercrops can thus be both positive and negative. The advantage of intercrops over sole crops lies in maximizing the complementarity and minimizing the competition between the components [8,24]. In simultaneous intercropping, aerial competition was found to decrease only by maintaining adequate plant spacing, thus the yield and quality advantage should not rely on overcrowding but only on the exploitation of beneficial belowground interactions [10]. In temperate and Mediterranean climates, the intercropping of grain legumes with cereals has proven to give higher forage yield over legume sole crops and better forage quality over cereal sole crops [17,25,26,27]. However, limited or no advantages have also been reported, which is probably due to the choice of companion crops, crop density, availability of resources, and environmental conditions [6,17,28,29].

In legume/cereal intercrop research, it is therefore crucial to understand whether the benefits at the root level, such as the complementarity in the use of water and N sources and the facilitation in mineral acquisition, can overcome the negative effect of the aboveground competition for light, or vice versa [30]. Above and belowground interactions are difficult to study separately, especially in field grown crops, and the latter are very time-consuming and pose technical challenges [31,32,33]. However, the positive contribution to aboveground yield of the interspecific interactions occurring at the root level has been demonstrated by root separation experiments [34,35,36]. Wang et al. [37] reported that the shoot and grain biomass of intercropped wheat and chickpea were higher when roots were free to intermingle, whereas Mariotti et al. [17] found that root interactions favored the shoot growth of cereals but decreased that of legumes, when wheat and barley were intercropped with lupin and vetch. These results highlight that the roots of intercropped cereals and legumes may also compete, which seems to be more pronounced at the initial growth stages and to depend on differences between companion species in the size of seeds and in the initial growth-rate of roots [30,38].

Investigations on belowground interactions are thus of huge interest in understanding the mechanisms underlying the agronomical and ecological advantages of intercrops over sole crops [9,39]. There are many aspects to consider, which range from changes in root size, architecture, and distribution in the soil profile, to changes in the efficiency in mineral uptake. These, in turn, can be modulated by either root exudation or changes in the abundance, composition and activity of soil microorganisms, or both. Most intercrop research has either neglected root investigations or limited measurements to a few parameters. These studies have demonstrated that interspecific root contact may reduce [17] or increase the root biomass of the companion species [40], or decrease the legume root biomass only [14], which is also dependent on the nitrogen supply [34]. Intercropping was found to change root distribution in the soil profile and stimulated lateral root growth [32,33,40]. Nodulation patterns in intercrops have rarely been investigated and contrasting results have been reported. Increased nodule initiation and growth were reported in chickpea/wheat, faba bean/wheat, and faba bean/maize intercrops [41,42,43,44], while increased nodule initiation but strong growth reduction was found in cowpea intercropped with maize [14]. Nodulation patterns in intercrops were found to change in relation to the mineral N supply in field bean/barley intercrops, but not in alfalfa/triticale intercrops [44,45]. Liu et al. [16] found that the roots of wheat intercropped with faba bean stimulated nodulation by increasing the exudation of flavonoids in the rhizosphere, but only at low N fertilizer levels. The intercropping of sugarcane with soybean promoted populations of nitrogen-fixing bacteria and dehydrogenase activity in soil [46]. On the other hand, the intercropping of wheat with faba bean favored mycorrhizal infection in cereal roots [47], thus supporting the uptake of water and P, Fe, and Zn, which are key elements for human and livestock nutrition [15,48].

To investigate the interactions occurring above and belowground between the partner species, we arranged a 1:1 field bean/triticale row-intercrop in growth boxes, in which the shoots of adjacent rows were always in contact, while the roots were separated (Aerial interaction) or free to intermingle (Full interaction). Field bean and triticale were chosen because of their wide use in livestock nutrition and their good yield and quality performance in intercrops [27,49]. A replacement design was adopted in order to prevent plant overcrowding confounding the response to interspecific interactions in intercrops. Two levels of N fertilizer were tested, whereas no P fertilizers were added.

The research aimed at demonstrating the existence of positive interactions, such as complementarity and facilitation, between the roots of field bean and triticale, and at assessing the contribution of these interactions to shoot biomass and N and P yield. Sole crops of both species were arranged similarly to intercrops to compare inter- and intraspecific competition between shoots, and to reveal intraspecific competition between root rows, which to the best of our knowledge, has not been taken into account in previous intercropping studies [17,34,38]. The effects of above and belowground interactions were assessed in both species in terms of i) shoot and root growth; ii) resource allocation to above and belowground plant parts and to reproductive structures; iii) root morphometry and functional traits; and iv) N and P uptake efficiency. The performance of the field bean/triticale intercrop over the respective sole crops was also estimated. Moreover, as microorganisms can have a role in facilitation by mobilizing the unavailable pool of nutrients, we also determined the activities of specific indicator enzymes.

## 2. Results

### 2.1. Intercrop Performance

In replacement intercrop designs, a Land Equivalent Ratio (LER) higher than 1 indicates that intercrops (IC) yield more than the cumulated sole crops (SC) on a land basis, while values lower than 1 indicate the opposite. In our research, the LERs calculated for the shoot biomass and N and P yields of shoots were always higher than 1 in the Full treatment and lower than 1 in the Aerial treatment. The only exception was for shoot N yield in Aerial-N120, thus demonstrating that root interactions played a primary role in advantaging intercrops over sole crops (Table 1). The biomass advantage was more pronounced with the low N input, whereas the N and P yield advantages with high N input, and the differences were less pronounced in the Aerial treatments.

The Competitive balance (Cb) index equals zero when intercropped species do not compete or have equal competitive abilities, while all the other values indicate that one species is less affected or benefits more from intercropping than the other. In our research, the Cb for shoot biomass showed positive values, indicating a greater advantage for field bean (Fb), especially with the lower N supply and in the Aerial treatments (Table 1). Triticale (T) was more competitive in terms of N accumulation, and Cb absolute values were higher with the high N supply and with Full treatments. Crop competitiveness in terms of P accumulation depended on the N level, with Fb being more competitive with a low N supply and T with a high N supply. It is worth noting that the competitive ability of triticale was always more pronounced when roots intermingled with those of field bean.

### 2.2. Field Bean

#### 2.2.1. Plant Growth

**Figure 1:**
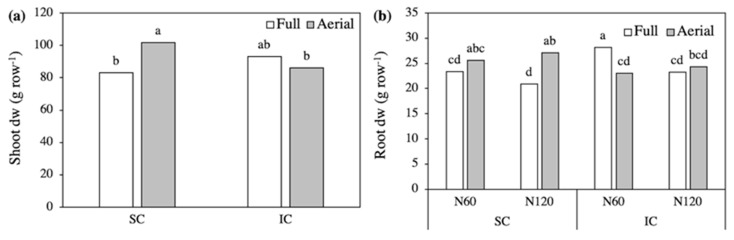
Shoot (**a**) and root (**b**) biomass of field bean, as affected by the Crop system × Row contact interaction (shoot) and by the Crop system × N level × Row contact interaction (root). SC, sole crop; IC, intercrop. Values are means of two N levels and three replicates (shoot) and of three replicates (root). In each figure, bars with different letters indicate that the corresponding means are significantly different at *P* < 0.05, Tukey’s test.

Analysis of variance revealed a significant second-order Crop system × Row contact interaction for the shoot biomass of field bean. The highest shoot biomass was recorded in the Aerial SC and the lowest in the Full SC, suggesting that, in Fb, shoot growth was negatively affected by intraspecific root competition and by interspecific shoot competition, however the latter seemed to slightly benefit from root contact (Figure 1a). The nitrogen level did not interact with the other treatments and, on average, shoot biomass was higher with the lower N supply, 94.4 g row^−1^ (N60) compared to 87.7 g row^−1^ (N120).

Shoot organs were affected differently by treatments. Significant Crop system × N level and Crop system × Row contact interactions were revealed for pod biomass and pod yield components. On the other hand, stem biomass was affected only by the Crop system × Row contact interaction, and leaf biomass did not change across treatments, measuring on average 19.5 g row^−1^. Pod biomass was markedly higher in the N60 IC and did not differ among the other treatments, which was essentially due to the higher pod number (Table 2). Accordingly, the partitioning into reproductive structures was significantly higher in the N60 IC than in the other treatments. The mean seed weight was not affected by any treatment and was, on average, 160 mg per seed. Stem biomass was highest in the Aerial SC and lowest in the Aerial IC, with intermediate and almost equal values in the Full treatments, while pod biomass and number were markedly lower in the Full SC than in all other treatments (Table 3). Finally, pods accounted for 43% of shoot biomass in IC and for approximately 36% in SC, without differences between row contacts.

The biomass of the entire root system changed in response to the third-order interaction. In SC, root biomass was lower in the Full than Aerial treatments, while in IC, it was higher in the Full treatment with N60 and did not differ between row contacts with N120 (Figure 1b). Accordingly, root biomass was highest in the N60-Full IC and the N120-Aerial SC, 28 and 27 g row^−1^, respectively. The root:shoot ratio was slightly higher in IC than in SC, 0.28 compared to 0.26, and was not significantly affected by the other treatments (data not reported).

#### 2.2.2. Root Morphometry

A significant Crop system × N level × Row contact interaction was revealed for lateral root biomass and for root morphometric and functional traits. The distribution of lateral roots in the soil profile changed according to the Crop System × Row contact × Soil depth interaction, without differences in response to the N level.

Tap root biomass was not affected by treatments and measured, on average, 8.5 g row^−1^. Lateral root biomass was approximately 16 g row^−1^ without differences among crop systems and N levels when roots were separated, but was by 45% higher in the N60-Full IC than in the N120-Full SC when roots were free to intermingle (Table 4).

On average, field bean had longer lateral roots in IC than in SC and, while in IC, root length increased with mineral N supply in both Full and Aerial treatments, in SC, the increase was significant only in the latter. The average diameter of lateral roots was slightly higher in Full than in Aerial SC, while the opposite occurred in IC, thus highlighting that the interaction with triticale led to the production of fine roots, especially with the high N supply. Root surface was markedly higher in IC than SC with corresponding treatments, with the greatest increases, 47%, recorded in the Full treatments (Table 4). On average, root surface was also higher in Aerial than in Full treatments and responded positively to N supply.

The contrasting response to treatments of the lateral root biomass and morphometric traits changed the root functional traits (Table 4). The Specific Root Length (SRL) was higher in IC than in SC with corresponding treatments and increased with the increase in N level. This thus highlighted that mineral N led to root elongation per unit biomass in Fb, independently of the contact with other roots. However, root contact also influenced the SRL, which was significantly lower in Full than in Aerial treatments except in the N120-IC, which recorded the highest SRL values. Root Tissue Density (RTD) is a measure of root porosity, which is used to express the energy investment per root unit. In contrast to SRL, the RTD of Fb was generally higher in SC than in IC, and the lowest values were found in the N120 Full treatment (Table 4).

**Figure 2:**
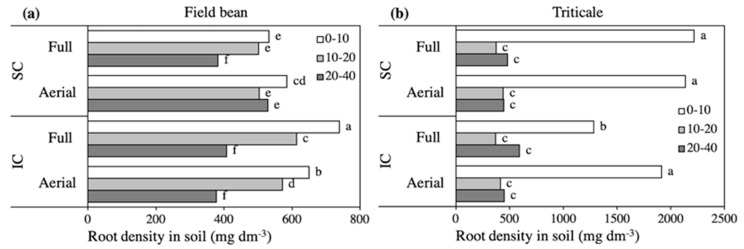
Root biomass density in soil of field bean (**a**) and triticale (**b**), as affected by the Crop system × Row contact × Soil depth interaction. SC, sole crop; IC, intercrop. Values are the mean of two nitrogen rates and three replicates. Within each figure, bars with different letters indicate that the corresponding means are significantly different at *P* < 0.05, Tukey’s test.

In all treatments the density of lateral roots in soil was higher in the upper 10-cm, however the distribution in the soil profile was greater even in sole crops than in intercrops, because of the marked increase in roots both in the 10 and 20 soil layers in the intercrops (Figure 2a). As a consequence, the partitioning of root biomass was 37-33-30% in SC, and 41-35-23% in IC in the 10, 20, and 40 cm layers, respectively, without significant differences between the Full and Aerial treatments in IC. In the 10-cm soil profile, the average diameter of roots was slightly lower in the Full IC (0.69) than in the other treatments, (0.76 mm), whereas in the two other layers it was 0.83 mm, again without significant differences among treatments (data not shown).

#### 2.2.3. Nodule Initiation and Growth

**Figure 3:**
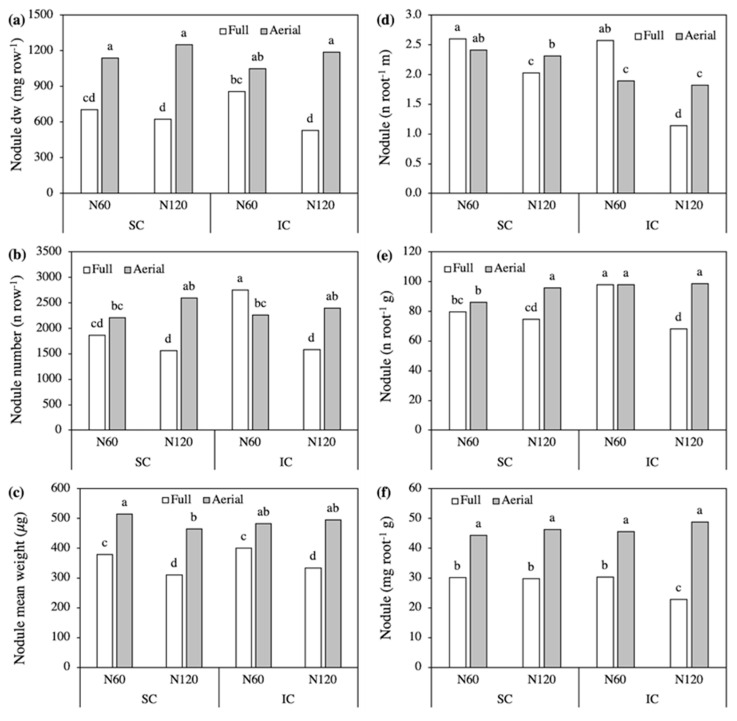
Nodule parameters (**a**–**c**) and nodule:root ratios (**d**–**f**) as affected by the Crop system × N level × Row contact interaction. SC, sole crop; IC, intercrop. Values are the mean of three replicates. In each figure, bars with different letters indicate that the corresponding means are significantly different at *P* < 0.05, Tukey’s test.

Treatments affected nodule initiation and growth differently with a significant third-order interaction in terms of biomass and number. Both in SC and IC, the overall biomass of nodules was markedly lower in Full than in Aerial treatments, demonstrating that intra and interspecific competition between roots were always detrimental to nodule growth (Figure 3a).

In Aerial treatments, nodule biomass was slightly, and not significantly, lower with the higher N supply. Conversely, in Full treatments nodule biomass was significantly higher with N60 than with N120 in IC and showed intermediate values without differing in response to N in SC. The high nodule biomass recorded in the N60-Full IC was associated with a huge increase in nodule number compared to the other Full treatments (Figure 3b). A slightly higher nodule number with N60 compared to N120 was also recorded in the Full SC, while the opposite occurring in the Aerial treatments. The mean nodule weight was not affected by crop system, and thus in both SC and IC it was higher in Aerial than in Full treatments and, while in the Aerial treatment it did not change significantly in response to the N level, in the Full treatment it was lower with the higher N supply (Figure 3c).

As the changes in nodule number and biomass could in part be a consequence of the size of the root system, we calculated the nodule density on root length and root biomass and also the nodule to root biomass ratio (Figure 3d–f). In Aerial treatments, the number of nodules per unit root length was higher in SC than IC without differences in response to N level, thus reflecting the enhancement in root elongation driven by aboveground competition with triticale (Figure 3d). In contrast, in Full treatments, the nodules on root length decreased markedly in response to high N supply, by 22% in SC and by 55% in IC, to which both the reduced nodulation and increased elongation of roots contributed. The number of nodules per unit of total root biomass was lower in SC than IC with N60, without differences between Full and Aerial treatments (Figure 3e). With N120, this ratio was significantly lower in Full treatments in both SC and IC, highlighting that high mineral N depressed nodule initiation compared to root biomass, but only in response to the competition between roots from adjacent rows. As the nodule:root weight ratio was always markedly higher in Aerial than in Full treatments, our results demonstrate that nodule growth was also impaired more than root growth by root competition, and the greatest reduction was recorded in the N120-Full-IC (Figure 3f).

#### 2.2.4. Nutrient Uptake 

In field bean, the Crop system, Row contact, N level, and Soil depth treatments did not significantly affect the N and P concentration and content of aerial and hypogeal plant parts. On average, the N concentration (mg g^−1^) decreased in the order: 36.1 in pods, 24.0 in lateral roots, 14.9 in stems+leaves, and 10.6 in tap roots. The P concentration (mg g^−1^) was 2.7 in pods, 1.5 in lateral roots, 1.3 in stems+leaves, and 0.9 in tap roots. A slight response to N level was observed in the N and P concentrations of shoots in that the supply of 120 kg N ha^−1^ increased the average N concentration of the shoot by 4% and its P concentration by 7%. This did not affect the shoot N and P contents, because of the slight decrease in biomass recorded with the higher N supply (data not reported). Accordingly, averaged over all treatments, the N content was 2120 mg row^−1^ in shoots and 473 mg row^−1^ in roots, while the P content was 171 and 31 mg row^−1^, respectively.

**Figure 4:**
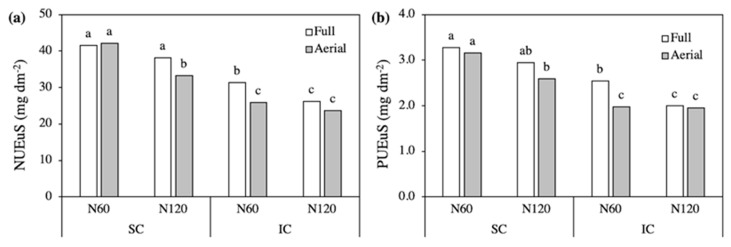
Nitrogen (**a**) and phosphorus (**b**) uptake efficiency per unit of root surface in field bean, as affected by the Crop system × N level × Row contact interaction. SC, sole crop; IC, intercrop. Values are the mean of three replicates. In each figure, bars with different letters indicate that the corresponding means are significantly different at *P* < 0.05, Tukey’s test.

Due to the huge differences in lateral root development, however, the amount of N and P taken up per unit of root surface (NUEuS and PUEuS) differed greatly among treatments. The NUEuS was higher in SC compared to IC, with the highest values recorded in N60 SC without differences between Full and Aerial treatments (Figure 4a). In the other combinations of treatments, in fact, NUEuS was always slightly higher in Full treatments. The PUEuS showed similar trends, always with lower values in IC than in SC except in the N60 Full treatment (Figure 4b).

### 2.3. Triticale

#### 2.3.1. Plant Growth

**Figure 5:**
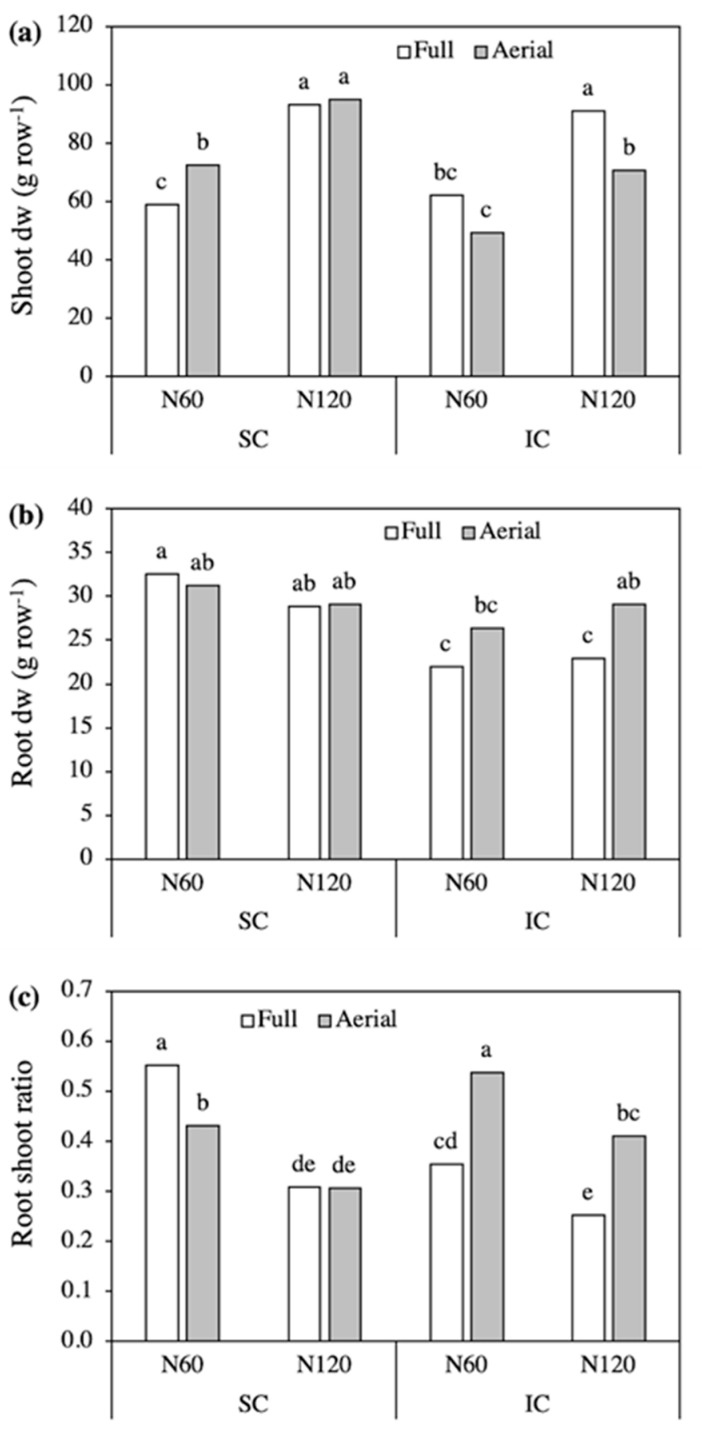
Shoot (**a**) and root (**b**) biomass and root:shoot ratio (**c**) of triticale, as affected by the Crop system × N level × Row contact interaction SC, sole crop; IC, intercrop. Values are means three replicates. In each figure, bars with different letters indicate that the corresponding means are significantly different at *P* < 0.05, Tukey’s test.

Analysis of variance revealed significant third-order Crop system × N level × Row contact interactions for the shoot and root biomass of triticale. The shoot biomass responded positively to the increase in N supply, with a higher responsiveness to N in Full than Aerial treatments, especially in SC (Figure 5a).

In addition, with corresponding N levels, shoot biomass did not differ significantly between SC and IC in the Full treatments, while it was markedly lower in IC than in SC in the Aerial treatments. The root biomass was not significantly affected by N level and row contact in SC (Figure 5b). Intercropping decreased root dry weight, however the differences in SC were only significant when roots of triticale and field bean were in contact. As the increase in N supply did not change root biomass but markedly increased shoot biomass, the root:shoot ratio decreased in all treatments with the supply of 120 kg N ha^−1^, with more pronounced differences in the Full SC (Figure 5c). The high root:shoot ratio recorded in the Aerial IC with both N levels was primarily a consequence of the reduced shoot growth.

The biomass of separate shoot organs, such as spike yield and its components, were affected differently by the Crop system × Row contact interaction and by the N level mean effect, whereas no significant third-order interactions were revealed. Averaged over N levels, the biomass of culms+leaves was lowest in the Aerial IC, while the spike biomass was highest in the Aerial SC and the Full IC, which was prompted by a higher number of spikes per row in the Aerial SC and by the higher number of spikelets per spike in the Full IC (Table 5). The different response of vegetative and spike biomass to treatments slightly increased the partitioning of shoot biomass to spikes in IC. Averaged over Crop system and Row contact, the supply of 120 kg N ha^−1^ increased the vegetative biomass by 37% and almost doubled spike biomass, thus increasing the partitioning of biomass to spikes by approximately four percentage points (Table 6). The huge increase in spike biomass in response to higher N supply was due to a slightly non significantly higher number of spikes per row and a higher number of complete spikelets per spike.

#### 2.3.2. Root Morphometry 

Analysis of variance revealed a significant Crop system × Row contact × Soil depth interaction for the density of roots in the soil profile, while the N level did not affect this interaction (Figure 2b). Conversely, root length and surface and functional traits changed according to the third-order Crop system × N level × Row contact interaction, without a significant interaction with Soil depth (Table 7). In all treatments, the average root diameter was 0.30 mm in the upper 0–10 cm of soil and 0.26 mm at deeper depths, and at least 98% of roots had a diameter lower or equal than 0.8 mm (data not shown).

The density of root biomass in soil was always markedly higher in the upper 10 cm, and the differences in response to treatments were significant only in this layer (Figure 2b). The interaction with Fb roots decreased the root density, which affected root partitioning in the soil profile by decreasing the proportion of roots in the upper layer and increasing it in the deep soil. As a result, the percentage partitioning was 57-17-26 in the 10, 20, and 40 cm layers, respectively, in the Full IC, and approximately 71-14-15 in the other treatments.

In triticale, root length was significantly higher in plants grown with only the Aerial contact compared to the corresponding Full treatments, and the differences were more pronounced in IC than in SC (Table 7). The increase in N level reduced root length in all treatments, with a percentage decrease of approximately 10% in the Aerial treatments of both crop systems; and 16 and 22% in the Full SC and Full IC, respectively. Root surface changed similarly to length in response to treatments, and lower values were recorded in the two Full IC and in the N120-Full SC (Table 7).

The Specific Root Length was always lower in Full than in corresponding Aerial treatments, which was primarily the consequence of reduced root elongation, and the highest SRL values were found in the N60 IC (Table 7). The Root Tissue Density changed less than SRL, and in IC was approximately 22% lower in Full than Aerial treatments without differences in response to N level (Table 7). In SC, conversely, RTD showed the lowest values in the N60 Aerial treatment, which was associated with the highest root surface.

#### 2.3.3. Nutrient Uptake

In triticale, the treatments influenced the N and P concentration and content of all plant parts, with patterns that differed between chemical elements and between roots and shoots. The culms, the leaves, and the spikes showed similar changes in response to treatments. Therefore, data were combined for the entire shoot. Plants intercropped with field bean showed higher N concentration in the shoots irrespective of the type of row contact, but differences were significant only with the supply of 120 kg N ha^−1^ (Table 8).

The shoot N content was generally higher in IC than in SC and increased with N supply but differed in response to row contact only in the N120 IC, being 44% higher in the Full than in the Aerial treatment. The shoot N content in the N120-Full IC was up to 200% higher than in the other treatments. The N concentration of roots changed only in response to Soil depth. Accordingly, the N content in response to Crop system, N level, and Row contact followed the root biomass patterns and is not reported. The N concentration was lowest in the upper 10-cm soil profile and highest in the intermediate one, whereas the N content was always markedly higher in the upper soil layer (Table 9).

In both triticale shoots and roots, the P concentration and content were affected by the third-order interaction. The P concentration of the shoot was markedly lower in the well fertilized SC, which could be interpreted as a dilution phenomenon dependent on the greater biomass (Table 10, Figure 5a). Due to the positive effect on growth, high N supply increased the P content of the shoots, with the highest increments (+61%) between the N60 and N120 Full-IC. In general, the P concentration of roots was higher in IC than SC and with N60 compared to N120, which could in part be due to the lower demands of smaller shoots (Table 10). The P concentration and content patterns in response to soil depth were similar to those of N, suggesting that high root density in the upper soil increased N and P contents but reduced their concentration, because of increased competition for mineral acquisition (Table 9).

**Figure 6:**
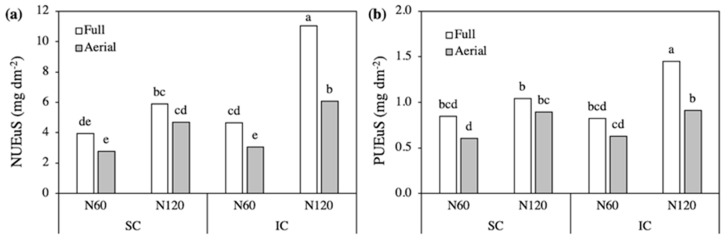
Nitrogen (NUEuS, **a**) and phosphorus (PUEuS, **b**) uptake efficiency per unit of root surface in triticale, as affected by the Crop system × N level × Row contact interaction. SC, sole crop; IC, intercrop. Values are the mean of three replicates. In each figure, bars with different letters indicate that the corresponding means are significantly different at *P* < 0.05, Tukey’s test.

The type of crop system did not influence the NUEuS and PUEuS of triticale with N60, while intercropping markedly increased N and P uptake efficiency with N120, especially with Full row contact (Figure 6). In addition, Full treatments always recorded higher values than the corresponding Aerial treatments. Accordingly, the amounts of both N and P taken up per unit of root surface were highest in the N120-Full IC. Specifically, NUEuS was 82% higher in the N120-Full IC than in the corresponding Aerial treatment, and 87% higher than in the N120-Full SC (Figure 6a). On the other hand, PUEuS was 59 and 39% higher, respectively (Figure 6b).

### 2.4. Soil Enzyme Activity

The activities of specific soil enzymes involved in the mobilization of nutrients were measured to estimate whether intercropping changed the soil environment of Full treatments. The three enzyme activities tested, FDA-ase, dehydrogenase, and phosphatase, showed intermediate rates in intercrops compared to the sole crops, with Fb SC showing the highest and T SC the lowest enzyme activities (Table 11). Significant differences were recorded between IC and Fb SC, but never between IC and T SC. In the field bean SC, the increase in N supply increased the activity of all tested enzymes, with significant differences in dehydrogenase and phosphatase activities (Table 11). In contrast, in triticale SC and in IC, all enzyme activities tended to decrease with the increase in N level, however the differences were not significant.

## 3. Discussion

The LERs obtained with the above and belowground plant parts that were free to interact (Full), were higher than 1 for all the parameters analyzed. This demonstrated that field bean and triticale intercropped in a row replacement design led to a greater yield than sole crops, thus complying with the primary goals of intercropping [17,18]. In addition, as the LER of plants intercropped without contact between roots from adjacent rows (Aerial) were almost always lower than 1, our results clearly demonstrated that belowground interactions were the primary drivers of biomass and N and P advantages and, thus, should be investigated in more depth for improved intercropping systems [4,9]. In this experiment, we feel that the worse performance of the Aerial intercrops could not be ascribed to the physical barrier placed between roots of adjacent rows, because, in the sole crops, the Aerial treatments yielded more than the Full treatments in terms of both shoot and root biomass. The LER values revealed that the biomass advantage of intercrops was greater with low N rates (60 kg ha^−1^) and that the N and P yields were higher with high N rates (120 kg ha^−1^). This suggests that intercropping could be profitable in both low and high input agriculture, giving primarily higher yields over than crops in low input agriculture, and better quality in terms of N and P concentration, in high input agriculture. The competitive balance indicated a slight advantage of field bean over triticale in terms of biomass production, while the opposite was reported by Sobkowicz [50] in additive field bean/triticale intercrops, which confirms that field bean suffers more than triticale when plant density is increased.

Higher N and P concentrations in the forage and grain compared to sole crops are a common feature of cereal/legume intercrops, as they can be achieved by niche differentiation and facilitation processes [1], but also by an increased proportion of legumes or lower biomass yield [51,52]. In our research, biomass was not reduced by intercropping and, therefore, the higher N and P concentration and content recorded in the Full intercropped triticale can be ascribed to improved uptake. As we adopted a replacement design, the higher intercrop yield did not rely on the increased plant density but should be interpreted as the positive balance of favorable interspecific interactions, such as facilitation and complementarity, over interspecific competition [1]. 

The comparison of the shoot biomass of sole cropped and intercropped field bean and triticale highlighted that, in both species and with both N levels, interspecific aboveground interactions were detrimental to shoot biomass, and that the negative effect of shoot competition was nullified when the roots were free to intermingle, thus highlighting that belowground interactions were favorable to both species. Previous studies have found that interspecific competition reduces the shoot growth of all species except vetch, when either barley or wheat were intercropped with lupin or vetch, but only the cereals benefitted from belowground interactions [17]. Similarly, Martin and Snaydon [34] reported that root interactions increased the competitiveness of barley towards field bean, progressively with growth stage and depending on the N supply. Finally, in maize/soybean intercrops, plant performance benefitted the most from root interactions only with narrow spacing and with a low N supply [36,53]. These findings highlight that the choice of intercrop design, species, and fertilizer level are key to successful intercropping.

The analysis of the single shoot organs showed that, in both field bean and triticale, the lower shoot biomass in response to interspecific aerial competition depended essentially on reduced stem/culm growth, as the leaves did not change and the reproductive biomass, pods/spikes, were not or only slightly reduced. In addition, in both species, the reproductive organs received the greatest benefits from interspecific root contact, so that the partitioning of shoot biomass into pods or spikes tended to be higher in intercrops than in sole crops. These results could be explained by a different balance between competition and benefits during the crop cycle, however it is not clear at which growth stage belowground interactions become positive. In support of our findings, Benincasa et al. [30] reported competition rather than complementarity between wheat and faba bean at early growth stages, with the latter being more aggressive. In field bean/triticale intercrops, triticale was the dominant crop at maturity [50], however during the vegetative phase, aboveground competition favored the field bean, belowground competition triticale, and no competition occurred when both plant parts interacted [38]. Similarly, Martin and Snaydon [34] found that barley exploited root interactions with field bean only later in the crop cycle. The different timing of aerial competition and belowground benefits should be further investigated, as this could question the advantage of intercropping for forage production.

Positive belowground interactions largely rely on the ability of mixed roots to avoid competing for the same soil space and mineral resources, and a greater proliferation of mixed root systems associated with a complementary redistribution in the soil profile is reported as a major effect of intercropping [1,9,33]. Wang et al. [13], however, reported that shading aided the proliferation of soybean roots intercropped with maize in the superficial soil. In our experiment, patterns of root distribution in the soil profile were in accordance, at least in part, with competition for space and resources. In fact, the proliferation of Fb lateral roots in the 0–10 and 10–20 cm soil layers occurred at the expense of triticale roots and was associated with a reduction in root diameter. Triticale thus formed a proportionally deeper root system, similarly to findings reported for barley intercropped with either pea or vetch [32,40].

In field bean, shoot and root biomass changed with similar patterns in response to treatments, with only a slightly higher partitioning of biomass to roots in IC compared to SC. In triticale, conversely, we found marked variations in the shoot to root ratio, revealing a great plasticity of biomass allocation in this crop, which is probably due to the different plant architecture of cereals and legumes. In fact, cereals are composed of a sum of nearly independent culm and root units, whereas legumes by branched but unique shoot and root systems. Low root:shoot ratios were found in triticale not only in the N120 SC, which is considered a typical response to high nutrient levels [54], but also in Full IC with both N60 and N120. The lower root biomass of triticale in Full IC treatments could thus be interpreted as a positive rather than a negative response. In addition, the greater partitioning of resources to shoots indirectly demonstrates the better uptake efficiency of T roots when intermingled with those of Fb. While root biomass showed similar results in the two species, between 23 and 28 g row^−1^ in Fb and 22–33 g row^−1^ in T, huge differences were recorded in root length and surface, which were, on average, 7 and 2.6 times greater in triticale. This did not seem to impair the uptake ability of field bean, as the uptake efficiency per unit root surface was approximately 6 and 3 times greater than in triticale in terms of N and P uptake, respectively.

Root morphometric traits responded differently to root neighboring. In triticale, both intra and interspecific row contacts were detrimental to root elongation, whereas in field bean, root length was only reduced by intraspecific root contact. Root traits also changed in contrasting ways in response to mineral N. In fact, high N supply slightly decreased root length in triticale but increased it in field bean, with marked increases prompted by interspecific root competition. We found that enhanced root elongation was associated in field bean with lower nodule initiation and growth, suggesting a shift from biological N_2_ fixation to competition for mineral N uptake when its availability in soil increased [44,55]. Reduced nodule biomass in fertilized intercrops was also found by Latati et al. [14] and Cardelli et al. [45], which questions the effectiveness of N source complementarity in cereal/legume intercrops [4,56,57]. However, as the N concentration and content increased in Full intercropped triticale without increasing soil scavenging and despite competition with Fb, it would seem that the N pool available for plant uptake increased when roots were in contact, probably through N release from decaying Fb roots and nodules [10]. On the other hand, in the N60 Full IC, the great increase in nodule number in Fb coupled with the increase in specific root length in triticale, suggest that niche complementarity for N source worked successfully with a low N input [16,36,43]. Patterns of shoot N concentration in both species and the root length and root:shoot ratio of triticale are consistent with an adequate N supply to both species [54,58]. In cereals intercropped with legumes, P uptake was found to be promoted by both exudates of legume roots and by microbial activity [14,35,37]. As no higher soil enzyme activity was observed in the intercrops, we believe that enhanced P uptake by the roots of triticale could be related to the great proliferation of field bean roots. In field bean, the N and P concentration and content remained fairly constant in all plant parts across treatments, despite the great changes in length and surface of roots, as with the nodule development. This demonstrates the great plasticity of Fb lateral roots in modulating uptake efficiency according to the plant’s needs, despite differences in nutrient availability and the competition with triticale. In contrast, Li et al. [11] reported no changes in root length and biomass in this species in response to localized nutrient patches.

Although a great promotion of soil enzyme activity in intercrops was not found, field bean sole crops showed higher soil enzyme activity compared to triticale sole crops, as also reported by Siczek et al. [12] in faba bean and wheat. According to Castellano and Dick [59] and Knauff et al. [60] this enhanced activity could be related to enzymes exuded by legume roots into the rhizosphere, and also to the development of large populations of soil microorganisms that metabolize the organic compounds exuded by roots. All enzyme activities increased with the N supply in field bean sole crops, thus proving that they are independent from nodule initiation and growth, which, conversely, decreased slightly. The benefits of field bean on soil biota were not evident in intercrops, probably because the triticale roots were much more abundant in the soil, thus exerting a greater influence on the soil biological activity. Only dehydrogenase activity, which is considered to be proportional to the biomass of soil microorganisms [61], was higher in the N60 intercrop than in the triticale sole crop, and this improvement corresponded to the highest number of nodules. In line with our findings, in sugarcane intercropped with legumes, Solanki et al. [46] reported that a wide range of nitrogen-fixing bacteria actively interacted with the soil, thus promoting the essential diazotrophic bacteria.

The root functional traits, Specific Root Length and Root Tissue Density, have rarely been examined in root interaction studies, but are widely used to describe the economy of root growth in response to soil conditions, and both parameters are negatively related to soil fertility and soil moisture, on a large-scale gradient [62,63]. However, Craine and Dybzinski [64] hypothesized those plants that are able to increase SRL should be more competitive in mineral uptake. In our research, SRL was 3–5 times higher in triticale than in field bean and showed a great plasticity in response to treatments. In both species, the SRL was higher when roots of adjacent rows were separated, demonstrating that both intra and interspecific root competition affected elongation more than biomass. High SRL in response to low N supply was found in triticale only when intercropped, highlighting that this species prompted root elongation without increasing biomass allocation to roots when in competition with field bean. In contrast to triticale, in field bean SRL increased with N supply, which was also observed in soybean in response to increased P availability [65]. Unlike SRL, RTD values were similar in the two species and were less affected by treatments. However, the generally lower RTD values recorded in Full treatments suggest that both species are able to compete for mineral uptake without an increasing investment in root dry matter. Interestingly, in field bean, root functional traits changed in response to aerial competition with triticale also without root contact.

Our study considered both intra and interspecific interactions, which revealed that in field bean, the primary negative determinant of shoot growth and pod yield was intraspecific belowground competition, followed in second order by the interspecific aerial competition with triticale, which occurred irrespectively of N level. The contact between Fb roots from adjacent rows was detrimental, especially to nodule initiation and growth and to root elongation, whereas root biomass changed in proportion to shoot biomass, as confirmed by the small changes in the root:shoot ratio and by the low Specific Root Length. Our findings contrast with those of Murphy and Dudley [66], who reported that soybean plants allocated more to roots in the presence of root neighbors, and are not in agreement with the theory of the “tragedy of the commons”, which predicts an exaggerated root production to the expense of fruit and seed yield when more individuals share the same soil [67]. In our research, in fact, the decrease in reproductive yield in response to root neighboring was not associated with a higher biomass allocation to roots. We believe that our findings provide new insights into the rationale for adopting wide row densities in Fb sole crops, which is not to reduce aerial competition for light, but to reduce the autotoxicity from root exudates and the competition for symbionts, the latter occurring despite artificial seed inoculation [68]. Given that we evaluated intraspecific competition between plant rows and not between individual plants as in most neighboring studies [69], the question remains as to why plants respond differently to individuals sharing the same row than to individuals in adjacent rows. We hypothesize that plants germinating close to each other in the same row interact for almost all their lifetime, thus developing an identity recognition. On the other hand, they recognize the roots from adjacent rows that they come into contact with later, as not kin [70,71].

Surprisingly, we found that field bean roots were markedly longer in the Aerial IC than in the Aerial SC, and thus changed in response to intra and interspecific aboveground interactions without root contact. In addition, while the root elongation prompted by root competition was associated with reduced nodulation, shoot interactions did not affect nodule initiation and growth, thus suggesting that these were independently regulated processes. Previous studies on the response of legumes to aboveground competition found that root biomass and biomass partitioning within the plant did not depend on the type of competition (inter or intra), and that shading inhibited root elongation in soybean intercropped with maize [13,66,69]. We cannot explain the mechanism by which the aerial contact between triticale and field bean prompted root elongation in Fb, however our findings suggest complex signaling between above and belowground plant parts and pose new challenges to plant neighboring investigations. Nevertheless, hypothesizing that shoots perceive neighbor identity similarly to roots [66], root elongation of field bean could be interpreted as a cue of impending competition for mineral N with triticale. Elongation did not lead to higher resource allocation for root construction, as demonstrated by the higher SRL in IC than in SC with corresponding treatments.

In triticale, unlike field bean, shoot growth responded primarily to N levels, and intra and interspecific interactions only modulated the positive response to the increasing N supply. In line with the characteristic of Poaceae to grow in dense populations, intraspecific competition was not observed between the aerial parts of plants in adjacent rows, while it was revealed between roots with a low N supply. Thus, in triticale, the lower shoot biomass coupled to a higher root:shoot ratio recorded in the Full compared to the Aerial sole crop with N60 is in agreement with the “tragedy of the commons”, which was driven by the competition of roots for mineral N [67].

## 4. Materials and Methods

### 4.1. Experimental Conditions

The research was carried out in 2018 at the experimental station of the Department of Agricultural, Food and Environmental Sciences of the University of Pisa, Italy, which is located at a distance of approximately 5 km from the sea (43°41′ N, 10°23′ E) and 1 m a.s.l. According to Köppen, the climate is classified as Csa, humid temperate with dry and hot summers. 

**Figure 7:**
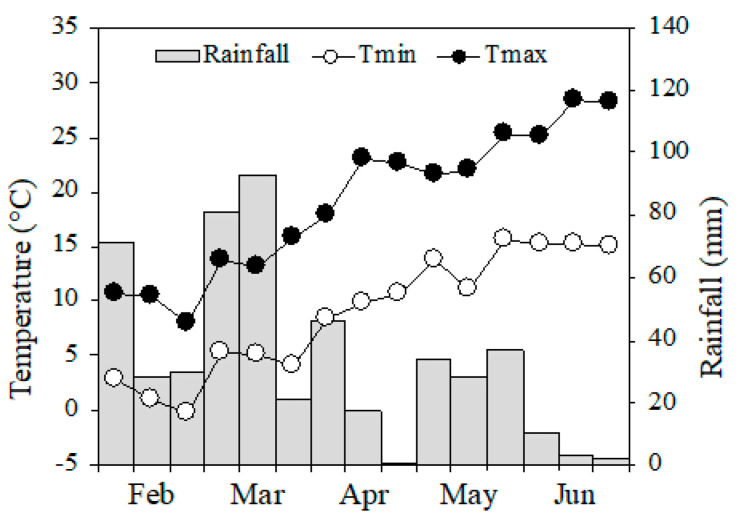
Air minimum and maximum temperatures and rainfall over the growing season 2018.

Meteorological data were obtained from a station located within 100 m of the trial site. Over the research period, the average daily temperature was 13.3 °C, the average maximum and minimum daily temperatures 18.2 and 8.3 °C, respectively, and the cumulated rainfall 427 mm (Figure 7).

### 4.2. Experiment Setup

The experiment was arranged with *Triticosecale* Wittmack cv. Trismart (triticale, T) and *Vicia faba* var. *minor* Beck cv. Vesuvio (field bean, Fb) grown as sole crops and intercrops in alternate rows within growth boxes that enable the roots of adjacent rows to be separated if necessary. The three crop plots, hereafter named crop systems, were field bean sole crop (Fb SC), triticale sole crop (T SC), and field bean/triticale intercrop (IC). All crop systems were split into two subplots with different levels of nitrogen supply, which were further split into two sub-subplots differing in the contact between plants in adjacent rows. The resulting arrangement was a split-split-plot design, with Crop system as the main plot, N level the subplot, and Row contact the sub-subplot, with three replicates.

**Figure 8:**
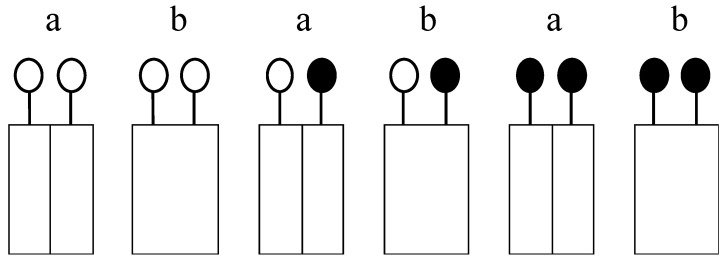
Schematic representation of the plant arrangement in sole crops and intercrops to give Aerial (**a**) and Full row contacts (**b**). Triticale (o); field bean (●).

**Figure 9:**
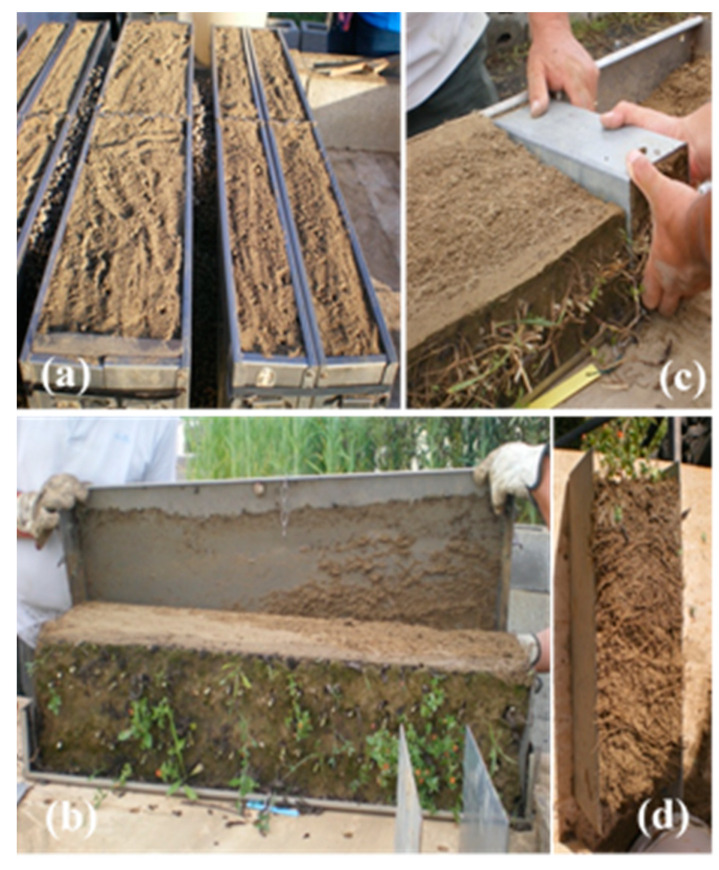
Growth boxes of Full (**a**, left) and Aerial (**b**, right) treatments; side front removal from a growth box at harvest (**b**); a soil column during (**c**) and after excavation (**d**).

The N levels corresponded to 60 (N60) and 120 (N120) kg N ha^−1^, which comprise low and medium rates, respectively, for cereal sole crops in the cultivation area. Both rates positively affected the yield parameters of field bean, although the higher rate decreased root length and nodule development [55,72]. The two types of contact between plants in adjacent rows were Full and Aerial. In the Full type, shoots and roots of plants in adjacent rows were free to interact, whereas in the Aerial type, a steel lamina completely separated the box in the middle, between the rows, so that interactions were limited to aerial parts (Figure 8 and Figure 9a). The aerial type was obtained using Donald’s separation technique [73], which has been successfully used in intercrops to study the nature of root and shoot interactions between the component crops [17,74,75].

For the experiment, 40 stainless-steel boxes with 1.0 m length, 0.2 m width, and 0.45 m depth, with large removable sides, were filled with 80 dm^3^ of soil. The physical and chemical properties of the soil were: 69.1% sand, 26.6% silt, and 4.3% clay (USDA method); pH 8.2 (soil:H_2_O 1:2.5), organic matter 17.3 g kg^−1^ (Walkley and Black method), total CaCO_3_ 5.4 g kg^−1^, total N 1.3 g kg^−1^ (Kjeldhal method), available P 7.4 mg kg^−1^ (Olsen method), available K 127 mg kg^−1^ (ammonium acetate method), conductivity 339 μS cm^−1^, and cation exchange capacity 8,0 meq 100 g^−1^ (BaCl2-TEA method).

### 4.3. Experiment Management

Plants were grown in 1 m long rows and each box contained two rows spaced 15 cm apart both for the sole crops and intercrops. This spacing was optimal for triticale, but narrow for field bean. Boxes were aligned on the long side, spaced 10 cm apart to maintain the row distance throughout crop plots and embedded in expanded clay to prevent daily fluctuations in soil temperature. To eliminate the border effect, four additional boxes were distributed at the beginning and end of the box arrangement and between crop system plots. The intercrop was a replacement design obtained by alternating rows of field bean and triticale.

Sowing took place on 9 February 2018, at densities of 30 seeds per linear meter of field bean and 60 seeds for triticale, corresponding to a seed density of 200 and 400 germinable seeds m^−2^, in the respective sole crops and of approximately 300 seeds m^−2^ in the intercrop. Field bean seeds were inoculated immediately before sowing with a specific commercial rhizobia inoculant using *Rhizobium leguminosarum* bv. *viciae*.

Just before sowing, the soil was fertilized with K_2_SO_4_ at a rate corresponding to 100 kg K ha^−1^ in all the treatments. Nitrogen fertilizer was applied in two rates, corresponding to 15 and 45 kg N ha^−1^ in N60 and to 30 and 90 kg N ha^−1^ in N120. The first rate was applied at sowing, as ammonium sulphate, and the second on 8 May, as urea. On this date, triticale was at the pseudo-stem erection stage (code 30, BBCH scale [76]) and field bean was at the flowering stage (63, BBCH scale). Phosphorus was not distributed so that root interactions in P mobilization and acquisition would be highlighted. To prevent competition for water, all boxes were watered daily to field capacity with a drip-irrigation system. Sporadic weeds were removed by hand, and the entire experimental block was covered with nets to exclude birds.

### 4.4. Shoot Harvest and Measurements

Plant harvest started on 4 June (123 DAS), in correspondence with the pod ripening stage of field bean (85–90, BBCH scale) and the flowering stage of triticale (61–69, BBCH scale). This harvest time was chosen in order to prevent root decay and leaf loss in field bean. Within each species, no appreciable differences in the phenological stage were recorded among the treatments.

Shoots were manually cut 2 cm above ground level. Plant height was measured, and the biomass of each species was divided into stems, leaves, and spikes or pods. The number of stems and pods, or spikes, was recorded. In field bean, the number of seeds was also determined, while this was not possible in triticale, due to the earlier growth stage. For dry weight determination, the aerial plant parts were oven-dried at 65 °C to constant weight.

The performance of the intercrop, in terms of shoot biomass and N and P yield per unit area, was expressed as the Land Equivalent Ratio (LER), as reported in Bedoussac and Justes (2011):

$$LER=\left(\frac{Y_{Fb\;IC}}{Y_{Fb\;SC}}+\frac{Y_{T\;IC}}{Y_{T\;SC}}\right)$$

where Y is the shoot biomass, or N and P content of the shoot, of either field bean (Fb) or triticale (T) grown in the intercrop (IC) or sole crop (SC). Data were calculated on a box basis. In a replacement intercrop design, LER values higher than 1 indicate that the intercrop yields more than the cumulated sole crops on a land basis, while values lower than 1 indicate the opposite.

The competitive ability of one species in intercropping, relative to the other, was measured by the Competitive Balance Index (Cb), as reported in Mariotti et al. [17]:

$$Cb=ln\left[\left(\frac{Y_{Fb\;IC}}{Y_{Fb\;SC}}\right)÷\left(\frac{Y_{T\;IC}}{Y_{T\;SC}}\right)\right]$$

where the symbols are the same used as those used for LER. According to Wilson [77], a Cb value of zero indicates no competition or equal competitive abilities, while any other value indicates that one species is less affected or has more of an advantage in intercropping than the other. In this equation, positive Cb values indicate that field bean has more of an advantage than triticale, while negative values highlight that triticale has the advantage.

### 4.5. Root Harvest and Measurements

After shoot harvest, the growth boxes were opened by removing one of the large sides and two soil columns were excavated, one from the mid and the other from the lateral position (Figure 9b–d). The dimensions of the soil columns were 10 × 10 × 40 cm in the Aerial treatments, and 10 × 20 × 40 cm in the Full treatments. Each column was further divided into three portions corresponding to the 0–10 (10), 10–20 (20) and 20–40 (40) cm soil layers. The two portions corresponding to the same layer of one box were merged into one sample and soaked overnight in water.

The next day, roots were extracted by gently washing them under a water flow and, to prevent the loss of root fragments, the washing water was filtered through a fine glass-fiber net (45 mesh cm^−2^). The washed roots were transferred to jars filled with water and stored at 4 °C until further cleaning, which was completed within one week under a magnifying glass. In roots of field bean, nodules were removed, counted, and weighed. A sub-sample of about 10 g from the root samples of all treatments and soil depths was cleaned to remove residual materials, such as weed roots, small woody fragments, and debris, and in the Full contact intercrop, to separate field bean and triticale roots based on the different morphological aspect and color of roots. The clean root sub-samples, residual materials, remainder of the root sample, and the nodules were gently dried between two filter papers to remove surface water, until no more water tracks remained on the paper, and weighed to obtain the fresh weight of each fraction [78]. The ratio of clean roots and the ratio of field bean and triticale in the subsamples were used to calculate the fresh weight of roots from each species in the washed root samples. The clean roots were further divided into two fractions: one was used for fresh and dry weight determination, after oven drying at 65 °C to constant weight, and for chemical analyses, while the other was used for image analysis.

### 4.6. Root Image Analysis

Root images were acquired with an Epson Perfection V800scanner equipped with a special lighting system to prevent shadows, and with a permanent calibration for WinRHIZO Regular software (Regent Instruments Inc., Québec, QC, Canada).

Before scanning, the roots of triticale were stained by immersion in Toluidine blue 0.05% for 5 min, and then rinsed with water to eliminate the color from the root surface. Staining was not necessary for field bean roots, which were of a dark brown color at this growth stage. The fresh root sub-sub-samples (about 0.1 g for triticale, and 1 g for field bean) were spread with minimal overlapping into 20 × 25 cm trays filled with a 0.5 cm layer of deionized water, and then scanned at 400 dpi. Fishing wires of 0.1 and 0.5 mm were added to the triticale and field bean roots, respectively, for the manual calibration of images. Roots with a diameter below 0.05 mm were not detected. After scanning, the fresh weight of the measured root sub-sub-samples was determined.

The root parameters length, surface, volume, and average diameter, were recorded with WinRHIZO separately for each soil layer. As root samples do not have a constant diameter, the surface and volume were obtained by adding the values obtained for the different diameter classes [79]. Parameters were measured separately for each soil layer. The root functional traits, Root Specific Length (RSL, m g^−1^ dw) and Root Tissue Density (RTD, g dw cm^−3^), were calculated [63] in order to estimate to what extent plants changed their resource use strategy when intercropped with and without contact between roots and in response to different levels of N supply. Both the functional traits were calculated on values per row.

### 4.7. Chemical Analyses

The dried leaves, stems, pods, and spikes were separately ground to powder (Cross Beater Mill, Retsch). The roots were milled at 18,000 rpm using a 0.5 mm Conidur distance-sieve (Ultra Centrifugal Mill ZM 200, Retsch). The nitrogen and phosphorus concentrations of the powders were determined by the modified Kjeldahl method and the ammonium–molybdophosphoric blue color method. The total N and P yields of separate plant parts were calculated by multiplying the N, or P, concentration per dry matter. The N and P uptake efficiency of roots (NUEuS and PUEuS) was estimated for both field bean and triticale as the amount of N and P absorbed per unit root surface, using the formula:

$$XUEuS=Y_{X}/RS$$

where X is either N or P, Y is the total plant content of N, or P, and RS is the root surface.

### 4.8. Soil Enzyme Activities

At plant harvest, soil samples were collected close to roots, sieved, and stored at 4 °C. Enzyme activities were assayed within one week of sampling. The hydrolysis rate of fluorescein diacetate (FDA-ase) was estimated as reported by Dick et al. [80], by determining the concentration of fluorescein released by FDA (µg g^−1^ 2 h^−1^) at 490 nm. The dehydrogenase activity (Deh) was determined by Tabatabai’s method [81] based on a colorimetric assay at 488 nm of 1,3,5 triphenylformazan (TPF) produced by the microorganism reduction of 2,3,5 triphenyltetrazolium chloride (TTC). Deh was expressed as µmol of TPF g^−1^ soil per hour. The alkaline phosphatase activity (Pal) was determined by the colorimetric assay based on the hydrolysis of p-nitrophenyl phosphate added to soil samples. This phosphate releases p-nitrophenol, which can be detected colorimetrically at 410 nm, and Pal was expressed as µmol of p-nitrophenol g^−1^ soil per hour [82]. All data were expressed on the oven-dry weight of soil, and the results were reported as the determination means of three replicates.

### 4.9. Statistical Analysis

Data on field bean and triticale were analyzed separately by analysis of variance (ANOVA), using JMP (v. 9.0.1, SAS 2010). For shoot parameters, the response to the Crop system, N level, Row contact, and their interactions was analyzed by arranging data in a split-split-plot design with three replicates, in which the crop systems (SC and IC) were designed as whole plots, the N levels (N60 and N120) as subplots, and the row contacts (Full and Aerial) as sub-subplots. For the root parameters, the treatment Soil depth was added as a nested sub-sub-subplot to the previous design, to test the effects of three soil depths (10, 20 and 40). For soil enzyme activities, only Full data were collected and analyzed, arranged in a split-plot design, with Crop System as the main plot, and N level as the sub-plot. Finally, the significance of the LER and Cb indices was tested by arranging data in a split-plot design with three replicates, in which N levels were the main plot and row contacts were the subplots. Significantly different means were separated at the 0.05 probability level by Tukey’s test [83].

## 5. Conclusions

Our study demonstrated that belowground interactions play a primary role in determining the aboveground biomass and the N and P yield of field bean/triticale intercrops. The advantage of intercrops over sole crops was in biomass production with the supply of 60 kg N ha^−1^, and in N and P yield with the supply of 120 kg N ha^−1^. In triticale, the benefits from interspecific root interactions relied primarily on the greater partitioning of resources to the shoot with the lower N supply and on the higher efficiency in N and P uptake per unit root surface with the higher N supply. In field bean, benefits of intercropping were essentially related to higher root biomass and length and also to higher nodulation with the lower N input. Conversely, our results demonstrated that field bean competed with triticale for mineral N, when its availability increased in the soil.

Thus, the observed intercrop benefits could be explained by the niche complementarity for N source only with the supply of 60 kg N ha^−1^. With the supply of 120 kg N ha^−1^, facilitation in N and P uptake could be hypothesized, which was probably related to the great proliferation of field bean roots, while specific soil enzyme activities were not increased or only minimally.

We found that both in field bean and triticale, intra and interspecific competition and N level affected root biomass and length with different patterns, which highlights that both root parameters should be of concern in intercropping studies.

## Figures and Tables

**Table 1:** Land Equivalent Ratio of intercrop versus sole crop and Competitive balance of field bean and triticale in the intercrop, as affected by the N level × Row contact interaction. For each parameter, values followed by the same letter within a column are not significantly different at *P* < 0.05, Tukey’s test.

N Level	Row Contact	Biomass	N Yield	P Yield
kg ha^−1^		Land Equivalent Ratio		
60	Full	1.12 a	1.16 b	1.06 a
	Aerial	0.77 c	0.85 c	0.83 b
120	Full	1.01 b	1.42 a	1.12 a
	Aerial	0.79 c	1.09 b	0.94 b
		Competitive balance		
60	Full	0.12 b	−0.12 b	0.20 a
	Aerial	0.23 a	0.01 a	0.18 a
120	Full	0.07 b	−0.65 c	−0.24 c
	Aerial	0.11 ab	−0.50 c	−0.05 b

**Table 2:** Biomass of pods and pod yield components in field bean, as affected by the Crop System × N level interaction. SC, sole crop; IC, intercrop. For each parameter, values followed by the same letter within a column are not significantly different at *P* < 0.05, Tukey’s test.

Crop System	N Level	Pods	Pods	Seeds	Pod/Shoot
	kg ha^−1^	g row^−1^	n row^−1^	n pod^−1^	%
SC	60	33.9 b	51.6 b	2.1 ab	36.0 b
	120	33.0 b	49.1 b	2.0 ab	36.3 b
IC	60	44.6 a	66.8 a	2.2 a	47.1 a
	120	33.0 b	53.0 b	1.9 b	39.0 b

**Table 3:** Biomass of stems and pods, and pod-yield components in field bean, as affected by the Crop System × Row contact interaction. SC, sole crop; IC, intercrop. For each parameter, values followed by the same letter within a column are not significantly different at *P* < 0.05, Tukey’s test.

Crop System	Row Contact	Stems	Pods	Pods	Seeds	Pod/Shoot
		g row^−1^	g row^−1^	n row^−1^	n pod^−1^	%
SC	Full	54.7 b	28.6 b	42.8 b	2.0 a	34.3 b
	Aerial	63.5 a	38.3 a	58.0 a	2.0 a	37.6 b
IC	Full	53.1 b	40.0 a	59.8 a	2.2 a	43.0 a
	Aerial	48.6 c	37.6 a	60.0 a	2.0 a	43.6 a

**Table 4:** Lateral root parameters, Specific Root Length (SRL) and Root Tissue Density (RTD) of field bean, as affected by the Crop system × N level × Row contact interaction. SC, sole crop; IC, intercrop. For each parameter, values followed by the same letter within a column are not significantly different at *P* < 0.05, Tukey’s test.

Crop System	N Level	Row Contact	Biomass	Length	Average Diameter	Surface	SRL	RTD
	kg ha^−1^		g row^−1^	m row^−1^	mm	dm^2^ row^−1^	m g^−1^	mg cm^−3^
SC	60	Full	15.4 b	716.8 d	0.80 ab	57.7 d	46.7 d	34.6 a
		Aerial	16.5 b	917.0 c	0.76 b	69.5 c	55.5 c	37.4 a
	120	Full	13.0 c	769.4 d	0.85 a	65.0 c	59.3 c	26.4 b
		Aerial	15.8 b	1124.2 b	0.75 bc	83.9 b	71.2 b	35.9 a
IC	60	Full	18.9 a	1069.4 b	0.79 ab	84.8 b	56.7 c	29.9 b
		Aerial	16.3 b	1192.7 b	0.83 a	99.5 a	73.1 b	28.0 b
	120	Full	16.3 b	1382.5 a	0.69 c	95.9 a	84.6 a	25.8 b
		Aerial	15.7 b	1318.3 a	0.77 b	101.8 a	84.2 a	26.7 b

**Table 5:** Biomass of shoot plant parts, and spike yield components in triticale, as affected by the Crop System × Row contact interaction. SC, sole crop; IC, intercrop. For each parameter, values followed by the same letter within a column are not significantly different at *P* < 0.05, Tukey’s test.

Crop System	Row Contact	Culms +Leaves	Spikes	Spikes	Spikelets	Spikes/ Shoot
		g row^−1^	g row^−1^	n row^−1^	n spike^−1^	%
SC	Full	59.5 a	16.6 b	40.5 b	20.8 a	21.8 b
	Aerial	65.0 a	18.7 a	48.8 a	17.4 ab	22.4 ab
IC	Full	57.5 ab	19.0 a	38.8 b	19.1 a	24.8 a
	Aerial	45.9 b	14.0 c	37.5 b	15.9 b	23.4 a

**Table 6:** Biomass of shoot plant parts, and spike yield components in triticale, as affected by the N level mean effect. For each parameter, values followed by the same letter within a column are not significantly different at *P* < 0.05, Tukey’s test.

N Level	Culms +Leaves	Spikes	Spikes	Spikelets	Spikes/ Shoot
kg ha^−1^	g row^−1^	g row^−1^	n row^−1^	n spike^−1^	%
60	48.0 b	12.6 b	39.6 a	16.9 b	20.8 b
120	65.9 a	21.6 a	43.1 a	19.7 a	24.7 a

**Table 7:** Root parameters, Specific Root Length (SRL), and Root Tissue Density (RTD) of triticale, as affected by the Crop system × N level × Row contact interaction. SC, sole crop; IC, intercrop. For each parameter, values followed by the same letter within a column are not significantly different at *P* < 0.05, Tukey’s test.

Crop System	N Level	Row Contact	Length	Surface	SRL	RTD
	kg ha^−1^		km row^−1^	dm^2^ row^−1^	m g^−1^	mg cm^−3^
SC	60	Full	7.3 cd	208.8 bcd	224.4 d	53.5 a
		Aerial	8.9 ab	298.3 a	283.7 b	38.9 b
	120	Full	6.1 ef	176.7 de	211.8 d	46.6 a
		Aerial	8.0 bc	217.8 bc	274.9 bc	46.8 a
IC	60	Full	6.8 de	192.7 cde	309.1 ab	38.2 b
		Aerial	9.1 a	235.9 b	342.8 a	47.2 a
	120	Full	5.3 f	161.5 e	232.5 cd	38.0 b
		Aerial	8.2 abc	219.0 bc	280.8 b	51.2 a

**Table 8:** Nitrogen concentration and content in the shoots of triticale, as affected by the Crop system × N level × Row contact interaction. SC, sole crop; IC, intercrop. For each parameter, values followed by the same letter within a column are not significantly different at *P* < 0.05, Tukey’s test.

Crop System	N Level	Row Contact	Nitrogen	
	kg ha^−1^		mg g^−1^	mg row^−1^
SC	60	Full	9.8 bc	577.8 c
		Aerial	8.6 c	621.8 c
	120	Full	9.1 c	849.7 bc
		Aerial	8.5 c	806.8 bc
IC	60	Full	11.4 b	708.6 c
		Aerial	10.7 bc	527.1 c
	120	Full	17.4 a	1582.0 a
		Aerial	15.5 a	1095.7 b

**Table 9:** Nitrogen and phosphorus concentration and content in the roots of triticale, as affected by the Soil depth mean effect. For each parameter, values followed by the same letter within a column are not significantly different at *P* < 0.05, Tukey’s test.

Soil Depth	Nitrogen		Phosphorus	
cm	mg g^−1^	mg row^−1^	mg g^−1^	mg row^−1^
0–10	6.7 c	12.4 a	1.0 b	1.8 a
10–20	9.9 a	4.0 b	1.2 a	0.5 b
20–30	9.0 b	4.4 b	1.1 ab	0.5 b

**Table 10:** Phosphorus concentration and content in the shoots and roots of triticale, as affected by the Crop system × N level × Row contact interaction. SC, sole crop; IC, intercrop. For each parameter, values followed by the same letter within a column are not significantly different at *P* < 0.05, Tukey’s test.

Crop System	N Level	Row Contact	Shoot		Root	
	kg ha^−1^		mg g^−1^	mg row^−1^	mg g^−1^	mg row^−1^
SC	60	Full	2.3 a	135.7 cd	1.32 ab	41.6 a
		Aerial	2.1 ab	152.4 bc	0.85 cd	27.3 bc
	120	Full	1.8 b	166.9 b	0.68 d	17.3 e
		Aerial	1.8 b	175.0 b	0.72 d	19.2 de
IC	60	Full	2.1 ab	129.3 cd	1.48 a	29.5 bc
		Aerial	2.3 a	114.7 d	1.39 a	33.2 ab
	120	Full	2.3 a	208.9 a	1.11 bc	25.1 cd
		Aerial	2.4 a	169.1 b	1.07 bc	30.7 bc

**Table 11:** FDA-ase, dehydrogenase and alkaline phosphatase in the soil of full treatments, as affected by the Crop system × N level interaction. SC, sole crop; IC, intercrop. Values followed by the same letter within a column are not significantly different at *P* < 0.05, Tukey’s test.

Crop System	N Level	FDA-Ase	Dehydrogenase	Phosphatase
	kg ha^−1^	Fluorescein µg g^−1^ 2h^−1^	TPF µg g^−1^ h^−1^	p-nitrophenol µg g^−1^ h^−1^
SC Fb	60	201 ab	3.90 b	286 b
	120	219 a	4.61 a	332 a
SC T	60	187 bc	3.09 d	263 bc
	120	170 c	2.73 d	260 c
IC	60	187 bc	3.60 bc	281 bc
	120	168 c	3.22 cd	268 bc

## References

[1] Duchene O., Vian J.F., Celette F. (2017). Intercropping with legume for agroecological cropping systems: Complementarity and facilitation processes and the importance of soil microorganisms. A review. Agric. Ecosyst. Environ..

[2] Bybee-Finley K., Ryan M. (2018). Advancing intercropping research and practices in industrialized agricultural landscapes. Agriculture.

[3] Wezel A., Casagrande M., Celette F., Vian J.F., Ferrer A., Peigné J. (2014). Agroecological practices for sustainable agriculture. A review. Agron. Sustain. Dev..

[4] Brooker R.W., Bennett A.E., Cong W.F., Daniell T.J., George T.S., Hallett P.D., Hawes C., Iannetta P.P.M., Jones H.G., Karley A.J. (2015). Improving intercropping: A synthesis of research in agronomy, plant physiology and ecology. New Phytol..

[5] Li L., Tilman D., Lambers H., Zhang F.-S. (2014). Plant diversity and overyielding: Insights from belowground facilitation of intercropping in agriculture. New Phytol..

[6] Hauggaard-Nielsen H., Jørnsgaard B., Kinane J., Jensen E.S. (2008). Grain legume–cereal intercropping: The practical application of diversity, competition and facilitation in arable and organic cropping systems. Renew. Agric. Food Syst..

[7] Cortois R., Schröder-Georgi T., Weigelt A., van der Putten W.H., De Deyn G.B. (2016). Plant–soil feedbacks: Role of plant functional group and plant traits. J. Ecol..

[8] Anil L., Park J., Phipps R.H., Miller F.A. (1998). Temperate intercropping of cereals for forage: A review of the potential for growth and utilization with particular reference to the UK. Grass Forage Sci..

[9] Ehrmann J., Ritz K. (2014). Plant: Soil interactions in temperate multi-cropping production systems. Plant Soil.

[10] Hauggaard-Nielsen H., Jensen E.S. (2005). Facilitative root interactions in intercrops. Plant Soil.

[11] Li H., Ma Q., Li H., Zhang F., Rengel Z., Shen J. (2014). Root morphological responses to localized nutrient supply differ among crop species with contrasting root traits. Plant Soil.

[12] Siczek A., Frąc M., Kalembasa S., Kalembasa D. (2018). Soil microbial activity of faba bean (*Vicia faba* L.) and wheat (*Triticum aestivum* L.) rhizosphere during growing season. Appl. Soil Ecol..

[13] Wang L., Zhou T., Cheng B., Du Y., Qin S., Gao Y., Xu M., Lu J., Liu T., Li S. (2020). Variable light condition improves root distribution shallowness and p uptake of soybean in maize/soybean relay strip intercropping system. Plants.

[14] Latati M., Blavet D., Alkama N., Laoufi H., Drevon J.J., Gérard F., Pansu M., Ounane S.M. (2014). The intercropping cowpea-maize improves soil phosphorus availability and maize yields in an alkaline soil. Plant Soil.

[15] Xue Y., Xia H., Christie P., Zhang Z., Li L., Tang C. (2016). Crop acquisition of phosphorus, iron and zinc from soil in cereal/legume intercropping systems: A critical review. Ann. Bot..

[16] Liu Y.C., Yin X.H., Zheng Y. (2020). Influences of intercropping and nitrogen supply on flavonoid exudation in wheat roots. J. Plant Nutr..

[17] Mariotti M., Masoni A., Ercoli L., Arduini I. (2009). Above- and below-ground competition between barley, wheat, lupin and vetch in a cereal and legume intercropping system. Grass Forage Sci..

[18] Lithourgidis A.S., Dordas C.A. (2010). Forage yield, growth rate, and nitrogen uptake of faba bean intercrops with wheat, barley, and rye in three seeding ratios. Crop Sci..

[19] Tosti G., Guiducci M. (2010). Durum wheat-faba bean temporary intercropping: Effects on nitrogen supply and wheat quality. Eur. J. Agron..

[20] Assefa G., Ledin I. (2001). Effect of variety, soil type and fertiliser on the establishment, growth, forage yield, quality and voluntary intake by cattle of oats and vetches cultivated in pure stands and mixtures. Anim. Feed Sci. Technol..

[21] Agegnehu G., Ghizaw A., Sinebo W. (2006). Yield performance and land-use efficiency of barley and faba bean mixed cropping in Ethiopian highlands. Eur. J. Agron..

[22] Strydhorst S.M., King J.R., Lopetinsky K.J., Harker K.N. (2008). Forage potential of intercropping barley with faba bean, lupin, or field pea. Agron. J..

[23] Klimek-Kopyra A., Kulig B., Oleksy A., Zając T. (2015). Agronomic performance of naked oat (*Avena nuda* L.) and faba bean intercropping. Chil. J. Agric. Res..

[24] Dhima K.V., Lithourgidis A.S., Vasilakoglou I.B., Dordas C.A. (2007). Competition indices of common vetch and cereal intercrops in two seeding ratio. Field Crop. Res..

[25] Danso S.K.A., Zapata F., Hardarson G., Fried M. (1987). Nitrogen fixation in fababeans as affected by plant population density in sole or intercropped systems with barley. Soil Biol. Biochem..

[26] Carr P.M., Horsley R.D., Poland W.W. (2004). Barley, oat, and cereal-pea mixtures as dryland forages in the northern great plains. Agron. J..

[27] Dordas C.A., Lithourgidis A.S. (2011). Growth, yield and nitrogen performance of faba bean intercrops with oat and triticale at varying seeding ratios. Grass Forage Sci..

[28] Trydeman Knudsen M., Hauggaard-Nielsen H., Jørnsgård B., Steen Jensen E. (2004). Comparison of interspecific competition and N use in pea–barley, faba bean–barley and lupin–barley intercrops grown at two temperate locations. J. Agric. Sci..

[29] Gooding M.J., Kasyanova E., Ruske R., Hauggaard-Nielsen H., Jensen E.S., Dahlmann C., Von Fragstein P., Dibet A., Corre-Hellou G., Crozat Y. (2007). Intercropping with pulses to concentrate nitrogen and sulphur in wheat. J. Agric. Sci..

[30] Benincasa P., Pace R., Tosti G., Tei F. (2012). Early interspecific interference in the wheat/faba bean (*Triticum aestivum/Vicia faba* ssp. minor) and rapeseed/squarrosum clover (*Brassica napus* var. *oleifera/Trifolium squarrosum*) intercrops. Ital. J. Agron..

[31] Heeraman D.A., Juma N.G. (1993). A comparison of minirhizotron, core and monolith methods for quantifying barley (*Hordeum vulgare* L.) and fababean (*Vicia faba* L.) root distribution. Plant Soil.

[32] Hauggaard-Nielsen H., Jensen E.S. (2001). Evaluating pea and barley cultivars for complementarity in intercropping at different levels of soil N availability. F. Crop. Res..

[33] Li L., Sun J., Zhang F., Guo T., Bao X., Smith F.A., Smith S.E. (2006). Root distribution and interactions between intercropped species. Oecologia.

[34] Martin M.P.L.D., Snaydon R.W. (1982). Root and Shoot interactions between barley and field beans when intercropped. J. Appl. Ecol..

[35] Li L., Tang C., Rengel Z., Zhang F. (2003). Chickpea facilitates phosphorus uptake by intercropped wheat from an organic phosphorus source. Plant Soil.

[36] Zhang X., Huang G., Bian X., Zhao Q. (2013). Effects of nitrogen fertilization and root interaction on the agronomic traits of intercropped maize, and the quantity of microorganisms and activity of enzymes in the rhizosphere. Plant Soil.

[37] Wang D., Marschner P., Solaiman Z., Rengel Z. (2007). Growth, P uptake and rhizosphere properties of intercropped wheat and chickpea in soil amended with iron phosphate or phytate. Soil Biol. Biochem..

[38] Sobkowicz P. (2005). Shoot and root competition between spring triticale and field beans during early growth. Acta Sci. Pol. Agric..

[39] Tosti G., Thorup-Kristensen K. (2010). Using coloured roots to study root interaction and competition in intercropped legumes and non-legumes. J. Plant Ecol..

[40] Ramirez-Garcia J., Martens H.J., Quemada M., Thorup-Kristensen K. (2015). Intercropping effect on root growth and nitrogen uptake at different nitrogen levels. J. Plant Ecol..

[41] Banik P., Midya A., Sarkar B.K., Ghose S.S. (2006). Wheat and chickpea intercropping systems in an additive series experiment: Advantages and weed smothering. Eur. J. Agron..

[42] Li Y.Y., Yu C.B., Cheng X., Li C.J., Sun J.H., Zhang F.S., Lambers H., Li L. (2009). Intercropping alleviates the inhibitory effect of N fertilization on nodulation and symbiotic N_2_ fixation of faba bean. Plant Soil.

[43] Bargaz A., Isaac M.E., Jensen E.S., Carlsson G. (2015). Intercropping of Faba Bean with wheat under low water availability promotes Faba Bean nodulation and root growth in deeper soil layers. Procedia Environ. Sci..

[44] Zhao Y., Liu X., Tong C., Wu Y. (2020). Effect of root interaction on nodulation and nitrogen fixation ability of alfalfa in the simulated alfalfa/triticale intercropping in pots. Sci. Rep..

[45] Cardelli R., Esnarriaga D.N., Mariotti M., Arduini I. Root Dynamics and Soil-Enzyme Activities in Field Bean/Barley Intercrops.

[46] Solanki M.K., Wang Z., Wang F.-Y., Li C.-N., Lan T.-J., Singh R.K., Singh P., Yang L.-T., Li Y.-R. (2017). Intercropping in sugarcane cultivation influenced the soil properties and enhanced the diversity of vital diazotrophic bacteria. Sugar Tec..

[47] Wahbi S., Prin Y., Thioulouse J., Sanguin H., Baudoin E., Maghraoui T., Oufdou K., Le Roux C., Galiana A., Hafidi M. (2016). Impact of wheat/faba bean mixed cropping or rotation systems on soil microbial functionalities. Front. Plant Sci..

[48] Ercoli L., Schüßler A., Arduini I., Pellegrino E. (2017). Strong increase of durum wheat iron and zinc content by field-inoculation with arbuscular mycorrhizal fungi at different soil nitrogen availabilities. Plant Soil.

[49] Jedel P.E., Helm J.H. (1993). Forage potential of pulse-cereal mixtures in central Alberta. Can. J. Plant Sci..

[50] Sobkowicz P. (2006). Competition between triticale (*Triticosecale* Witt.) and field beans (*Vicia faba* var. *minor* L.) in additive intercrops. Plant Soil Environ..

[51] Mariotti M., Masoni A., Ercoli L., Arduini I. (2012). Optimizing forage yield of durum wheat/field bean intercropping through N fertilization and row ratio. Grass Forage Sci..

[52] Neugschwandtner R.W., Kaul H.P. (2015). Nitrogen uptake, use and utilization efficiency by oat-pea intercrops. Field Crop. Res..

[53] Yang F., Liao D., Wu X., Gao R., Fan Y., Raza M.A., Wang X., Yong T., Liu W., Liu J. (2017). Effect of aboveground and belowground interactions on the intercrop yields in maize-soybean relay intercropping systems. Field Crop. Res..

[54] Garnett T., Conn V., Kaiser B.N. (2009). Root based approaches to improving nitrogen use efficiency in plants. Plant Cell Environ..

[55] Pampana S., Masoni A., Mariotti M., Ercoli L., Arduini I. (2018). Nitrogen fixation of grain legumes differs in response to nitrogen fertilisation. Exp. Agric..

[56] Hauggaard-Nielsen H., Ambus P., Jensen E.S. (2001). Interspecific competition, N use and interference with weeds in pea-barley intercropping. Field Crop. Res..

[57] Corre-Hellou G., Fustec J., Crozat Y. (2006). Interspecific competition for soil N and its interaction with N_2_ fixation, leaf expansion and crop growth in pea-barley intercrops. Plant Soil.

[58] Morell F.J., Cantero-Martínez C., Álvaro-Fuentes J., Lampurlanés J. (2011). Root growth of barley as affected by tillage systems and nitrogen fertilization in a semiarid Mediterranean agroecosystem. Agron. J..

[59] Castellano S.D., Dick R.P. (1991). Cropping and sulfur fertilization influence on sulfur transformations in soil. Soil Sci. Soc. Am. J..

[60] Knauff U., Schulz M., Scherer H.W. (2003). Arylsufatase activity in the rhizosphere and roots of different crop species. Eur. J. Agron..

[61] Chu H., Lin X., Fujii T., Morimoto S., Yagi K., Hu J., Zhang J. (2007). Soil microbial biomass, dehydrogenase activity, bacterial community structure in response to long-term fertilizer management. Soil Biol. Biochem..

[62] Krishnamurthy L., Ito O., Johansen C., Saxena N.P. (1998). Length to weight ratio of chickpea roots under progressively receding soil moisture conditions in a Vertisol. Field Crop. Res..

[63] Kramer-Walter K.R., Bellingham P.J., Millar T.R., Smissen R.D., Richardson S.J., Laughlin D.C. (2016). Root traits are multidimensional: Specific root length is independent from root tissue density and the plant economic spectrum. J. Ecol..

[64] Craine J.M., Dybzinski R. (2013). Mechanisms of plant competition for nutrients, water and light. Funct. Ecol..

[65] Watt M., Evans J.R. (2003). Phosphorus acquisition from soil by white lupin (*Lupinus albus* L.) and soybean (*Glycine max* L.), species with contrasting root development. Plant Soil.

[66] Murphy G.P., Dudley S.A. (2007). Above- and below-ground competition cues elicit independent responses. J. Ecol..

[67] Gersani M., Brown J.S., O’Brien E.E., Maina G.M., Abramsky Z. (2001). Tragedy of the commons as a result of root competition. J. Ecol..

[68] Asaduzzaman M., Asao T. (2012). Autotoxicity in beans and their allelochemicals. Sci. Hortic. (Amsterdam).

[69] Jacoby R., Peukert M., Succurro A., Koprivova A., Kopriva S. (2017). The role of soil microorganisms in plant mineral nutrition—current knowledge and future directions. Front. Plant Sci..

[70] Milla R., Forero D.M., Escudero A., Iriondo J.M. (2009). Growing with siblings: A common ground for cooperation or for fiercer competition among plants?. Proc. R. Soc. B Biol. Sci..

[71] Chen B.J.W., During H.J., Anten N.P.R. (2012). Detect thy neighbor: Identity recognition at the root level in plants. Plant Sci..

[72] Ercoli L., Arduini I., Mariotti M., Lulli L., Masoni A. (2012). Management of sulphur fertiliser to improve durum wheat production and minimise S leaching. Eur. J. Agron..

[73] Donald C.M. (1958). The interaction of competition for light. Aust. J. Agric. Res..

[74] Tofinga M.P., Paolini R., Snaydon R.W. (1993). A study of root and shoot interactions between cereals and peas in mixtures. J. Agric. Sci..

[75] Thorsted M.D., Weiner J., Olesen J.E. (2006). Above- and below-ground competition between intercropped winter wheat Triticum aestivum and white clover Trifolium repens. J. Appl. Ecol..

[76] Meier U. (2001). Growth Stages of Mono and Dicotyledonous Plants.

[77] Wilson J.B. (1988). Shoot competition and root competition. J. Appl. Ecol..

[78] Birouste M., Zamora-Ledezma E., Bossard C., Pérez-Ramos I.M., Roumet C. (2014). Measurement of fine root tissue density: A comparison of three methods reveals the potential of root dry matter content. Plant Soil.

[79] Rose L. (2017). Pitfalls in root trait calculations: How ignoring diameter heterogeneity can lead to overestimation of functional traits. Front. Plant Sci..

[80] Dick R.P., Breakwell D.P., Turco R.F., Doran J., Jones A. (1997). Soil Enzyme Activities and Biodiversity Measurements as Integrative Microbiological Indicators. Methods for Assessing Soil Quality.

[81] Tabatabai M.A., Wollum R.W., Art W., Angle S., Bottomley P., Bezdicek D., Smith S., Tabatabai A. (1994). Soil Enzymes. Methods of Soil Analysis Part 2: Microbiological and Biochemical Properties.

[82] Eivazi F., Tabatabai M.A. (1977). Phosphatases in soils. Soil Biol. Biochem..

[83] Booth G.D., Steel R.G.D., Torrie J.H. (1997). Principles and Procedures of Statistics: A Biometrical Approach.

